# Quantification of Lung Damage in an Elastase-Induced Mouse Model of Emphysema

**DOI:** 10.1155/2012/734734

**Published:** 2012-11-08

**Authors:** Arrate Muñoz-Barrutia, Mario Ceresa, Xabier Artaechevarria, Luis M. Montuenga, Carlos Ortiz-de-Solorzano

**Affiliations:** ^1^Cancer Imaging Laboratory, Center for Applied Medical Research (CIMA), University of Navarra, Avenida Pio XII 55, 31008 Pamplona, Spain; ^2^Biomarkers Laboratory, Center for Applied Medical Research (CIMA), University of Navarra, Avenida Pio XII 55, 31008 Pamplona, Spain

## Abstract

*Objective*. To define the sensitivity of microcomputed tomography- (micro-CT-) derived descriptors for the quantification of lung damage caused by elastase instillation. *Materials and Methods*. The lungs of 30 elastase treated and 30 control A/J mice were analyzed 1, 6, 12, and 24 hours and 7 and 17 days after elastase instillation using (i) breath-hold-gated micro-CT, (ii) pulmonary function tests (PFTs), (iii) RT-PCR for RNA cytokine expression, and (iv) histomorphometry. For the latter, an automatic, parallel software toolset was implemented that computes the airspace enlargement descriptors: mean linear intercept (*L*_*m*_) and weighted means of airspace diameters (*D*_0_, *D*_1_, and *D*_2_). A Support Vector Classifier was trained and tested based on three nonhistological descriptors using *D*_2_ as ground truth. *Results*. *D*_2_ detected statistically significant differences (*P* < 0.01) between the groups at all time points. Furthermore, *D*_2_ at 1 hour (24 hours) was significantly lower (*P* < 0.01) than *D*_2_ at 24 hours (7 days). The classifier trained on the micro-CT-derived descriptors achieves an area under the curve (AUC) of 0.95 well above the others (PFTS AUC = 0.71; cytokine AUC = 0.88). *Conclusion*. Micro-CT-derived descriptors are more sensitive than the other methods compared, to detect *in vivo* early signs of the disease.

## 1. Introduction

Chronic obstructive pulmonary disease (COPD) is a complex and heterogeneous disease. Traditionally, two phenotypes of COPD have been described: obstructive bronchitis and pulmonary emphysema. Because of current smoking trends and progressive aging of the world population, an increase in COPD prevalence and related mortality is expected in the coming decades [1]. Emphysema is defined pathologically as the permanent enlargement of the airspaces distal to the terminal bronchioles, accompanied by destruction of their walls, without obvious fibrosis [2, 3]. At the molecular level, emphysema is an inflammatory-driven process caused by the enzymatic destruction of lung elastin and collagen by neutrophil and macrophage elastase [4]. The process is in most cases induced by cigarette smoking [5]. 

Animal models are key tools to study the disease. Probably the most widely extended one is the mouse model of elastase-induced emphysema, due to its simplicity and low cost [6]. Efficient and sensitive quantification of the injury is needed for the characterization of the disease and the assessment of therapeutic interventions on this model. In this paper, we describe efficient and sensitive methods to quantify histologically airspace enlargement *ex vivo*. This can in turn be used as an appropriate reference gold standard for the comparison and evaluation of descriptors extracted from micro-CT and other non-radiological *in vivo* techniques. 

**Figure 1:**
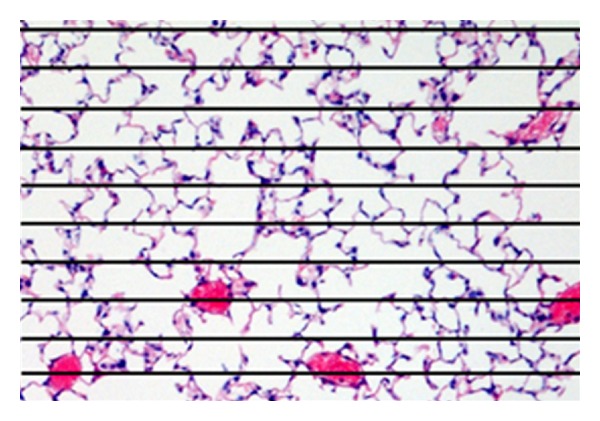
Calculation of the mean linear intercept (*L*_*m*_). A transparent sheet with 10 equally distributed horizontal lines is laid over the printed digitized image of an H&E-stained section. A transparent sheet with 11 equally distributed vertical lines is used thereafter (not shown). For each line, the intercepts with the tissue structures are counted. *L*_*m*_ is calculated as the ratio between the product of the number of times the traverses are placed on the lung and the length of the traverses, and the sum of all the intercepts.

The mean linear intercept (*L*_*m*_) [7] is a stereological metric established in the early sixties, commonly used to quantify emphysema in histological samples. *L*_*m*_ is perceived as an index of airspace size, although formally is a measurement of surface area-to-volume ratio. Computing *L*_*m*_ requires counting linear intercepts (see Figure 1), which is a relatively simple manual task [8]. Nevertheless, it cannot be equated with calculating the airspace size without knowledge of the shapes. Weibel et al. [9] illustrate this problem by comparing the *L*_*m*_ calculated both in a sphere and an ellipsoid of equal volumes. Both objects differ in their surface area, which is larger in the case of the ellipsoid. In consequence, measuring linear intercepts from random cross sections of both objects will yield a volume (*V*) to surface (*S*) ratio (*L*_*m*_ ∝ *V*/*S*) for the ellipsoid lower than for the sphere. Indeed, *L*_*m*_ underestimates the severity of emphysema in heterogeneous samples (i.e., samples containing many small airspaces surrounding a few enlarged ones).

To correct this problem, while taking advantage of the computational power of modern computers, Parameswaran et al. [10] presented three alternative measurements (*D*_0_, *D*_1_, and *D*_2_) based on the moments of the airspace equivalent diameters. When high moments are used, the largest airspaces are weighted more heavily than smaller ones. Therefore, *D*_2_ should be more accurate and robust than *L*_*m*_ to quantify heterogeneous, mild emphysema. The authors tested their hypothesis (i.e., shape dependence of *L*_*m*_ and its inability to detect airspace enlargement on heterogeneous samples) on both synthetic images and a few lung parenchyma images. Following this approach, Jacob et al. in [11] showed that *D*_2_ finds significantly more pronounced separation between the control and smoke-exposed mice (2–4 cigarettes/day, 6 days/week, 24 weeks) than *L*_*m*_.

Here, we present a fully automatic and parallel toolset that calculates the above-described metrics (*L*_*m*_, *D*_0_, *D*_1_, and *D*_2_) on entire histological sections at reasonable speed. We then used this in-house-developed software to quantify the emphysema in a large study of elastase-induced emphysema mouse model [12], starting at very early stages.

**Figure 2:**
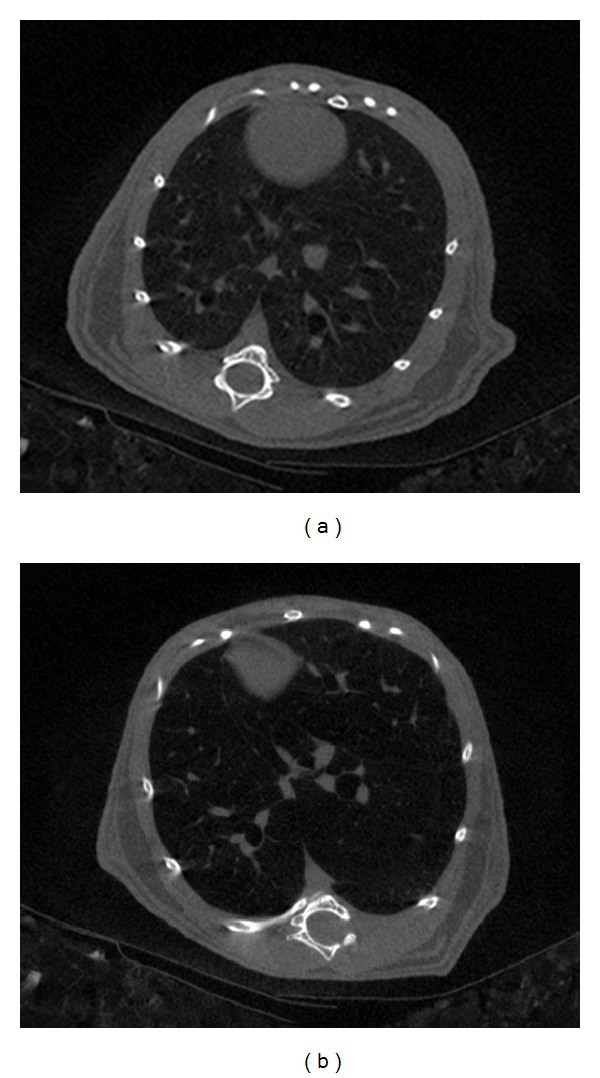
Sample transversal mouse lung micro-CT slices 17 days after instillation. (a) Control. (b) Elastase treated. Both slices are calibrated in Hounsfield units and displayed with a 2500 window centered at a level of zero. Note how the lung of the elastase-treated animal appears darker than the control lung.

Although histomorphometry is considered the most accurate method for morphological disease characterization, it is an *ex vivo* technique. There are several other techniques that can be used to characterize lung injury and emphysema in mice *in vivo*. This is of tremendous value to assess, for instance, the efficacy of new drugs or therapeutic interventions. Among them, micro-computed X-ray tomography (micro-CT) is an especially appropriate modality for lung imaging (Table 1) [13]. Using micro-CT, moderate to severe forms of emphysema can be easily detected in elastase-treated mice, as the lung parenchyma appears darker than in nontreated mice (see Figure 2). Pulmonary function tests (PFTs) provide physiological data about lung function and their characteristic parameters (Resistance and Compliance) [12]. Finally, the inflammatory response of the lungs to the damage caused by the insult can be evaluated by looking at protein or RNA cytokine levels, using blood plasma or lung tissue extracts. The information provided by these methods is complementary. 

In this study, we train a classifier to evaluate and compare the sensitivity of micro-CT density-based-derived descriptors and other non-radiological techniques (i.e., single compartment model PFTs, and RNA inflammatory cytokine levels) to detect elastase-induced lung damage. The histomorphometrical parameter *D*_2_ is used as reference value because is a generalized, shape-independent quantitative descriptor of airspace dimensions, well suited for automated computation. 

The paper structure is as follows. In the Materials and Methods, we describe the animal preparation, the PFTs and micro-CT imaging, and the sample preparation for RNA cytokine levels measurement and histology. We also present the image acquisition, the image analysis pipeline, and the statistical analysis of the histology data. Besides, we describe the classifiers that we used and how they were trained. The next section presents the results on the sensitivity of the histomorphometric descriptors to detect differences between the control and elastase-treated groups a few hours after the insult, and to study disease progression. Finally, we report on the ability of the rest of descriptors to adequately classify the animals using the histology as reference. The paper ends with a discussion and conclusions. 

## 2. Materials and Methods

### 2.1. Animal Preparation and *In Vivo* Tests

#### 2.1.1. Animal Preparation

All experimental protocols involving animal manipulation were approved by the University of Navarra Experimentation Ethics Committee. Sixty, 11-week-old mice were equally distributed into a control and a treatment groups. Treated mice were intratracheally instilled with 6 units per 30 g of porcine pancreatic elastase (PPE, EC134GI, EPC, MI, USA), as described in a previously published protocol [14]. Control animals were instilled with a saline solution. Five animals of each group were sampled at hours 1, 6, 12, and 24 and days 7 and 17 after treatment. At each time point, all animals underwent micro-CT thoracic imaging and pulmonary function tests. Then, they were sacrificed and their lungs collected for histomorphometry and cytokine measurements (RNA and protein).

#### 2.1.2. Pulmonary Function Tests

Animals were anesthetized, intratracheally cannulated, and connected to a Flexivent rodent ventilator set (Scireq, Montreal, QC, Canada) set at a rate of 200 breaths/min and a tidal volume of 10 mL/kg. Lung resistance (*R*) and compliance (*C*) values were measured using a single-frequency-forced oscillation, fitting the measured data to a single compartment model [15]. All measurements were repeated three times.

#### 2.1.3. Breath-Hold-Gated Micro-CT Imaging

The anesthetized, artificially ventilated mice were scanned using an X-ray micro-computed tomograph (Micro-CAT II, Siemens Pre-Clinical Solutions, Knoxville, TN, USA). Parameters used for the micro-CT image acquisition are those given in Table 2. Seven hundred micro-CT projections were acquired during 650 ms isopressure breath holds at 12 cm H_2_O. Two normal breathing cycles were induced between breath holds. A total lung capacity perturbation was performed every 20 breath holds to prevent atelectasis. 

The reconstructed three-dimensional images had a total of 640 slices with isotropic 46 *μ*m voxel size and 1024 × 1024 pixels per slice. The scanning time was approximately 30 minutes. The estimated X-ray dosage was 71.6 cGy/exam (Siemens Pre-Clinical Solutions, Knoxville, TN, USA). A water phantom was used to calibrate the image to Hounsfield units (HU). 

#### 2.1.4. Micro-CT Image Analysis

First, the lungs were automatically delineated in the 3D micro-CT images using an existing segmentation method [16]. Then, the airways were segmented and reconstructed using a fast and robust algorithm developed by us [17]. The algorithm is based on a propagating fast marching wavefront that divides the tree into segments as it grows. A number of specific rules were applied after every iteration of the front propagation to avoid unwanted leakage into the parenchyma. From the segmented airways, the right and left radius measurements of the mainstem bronchi (RMBR and LMBR, resp.) were computed. The airways were removed from the lung volume before quantification. Two other parenchymal injury descriptors were also computed: mean lung voxel intensity (MLVI) and relative volume below −900 HU (VBT). This threshold was selected because intensity values below −900 HU are rare in scans of healthy mice (the VBT is less than 5% of the total lung volume in all healthy animals of any age).

### 2.2. Sample Preparation for RNA Cytokine Expression and Histology

Mice were anesthetized and then sacrificed by exsanguination. Next, the lungs were fixed at a constant pressure of 20 cm H_2_O. RNA was extracted from the accessory lobe and then qRT-PCR was performed with an Applied Biosystems 7900HT Fast Real-Time PCR System. Four immune-modulatory cytokines and chemokines involved in lung inflammatory response [18] were analyzed, namely, interleukin 6 (IL6), immune protein 10 (IP10), keratinocyte chemoattractant (KC), and monocyte chemoattractant protein 1 (MCP1). Beta-2 microglobulin (B2M) was used as the endogenous control gene for the experiments. 

Three paraffin blocks from different lobules of each lung were created and reserved for histological analysis. Three nonconsecutive sections per block were cut and stained with Hematoxylin and eosin (H&E), resulting in a total of nine slides per mouse, each containing two lobe pieces. The total number of sections acquired and analyzed was 1080.

### 2.3. Histological Image Analysis

#### 2.3.1. Image Acquisition

**Figure 3:**
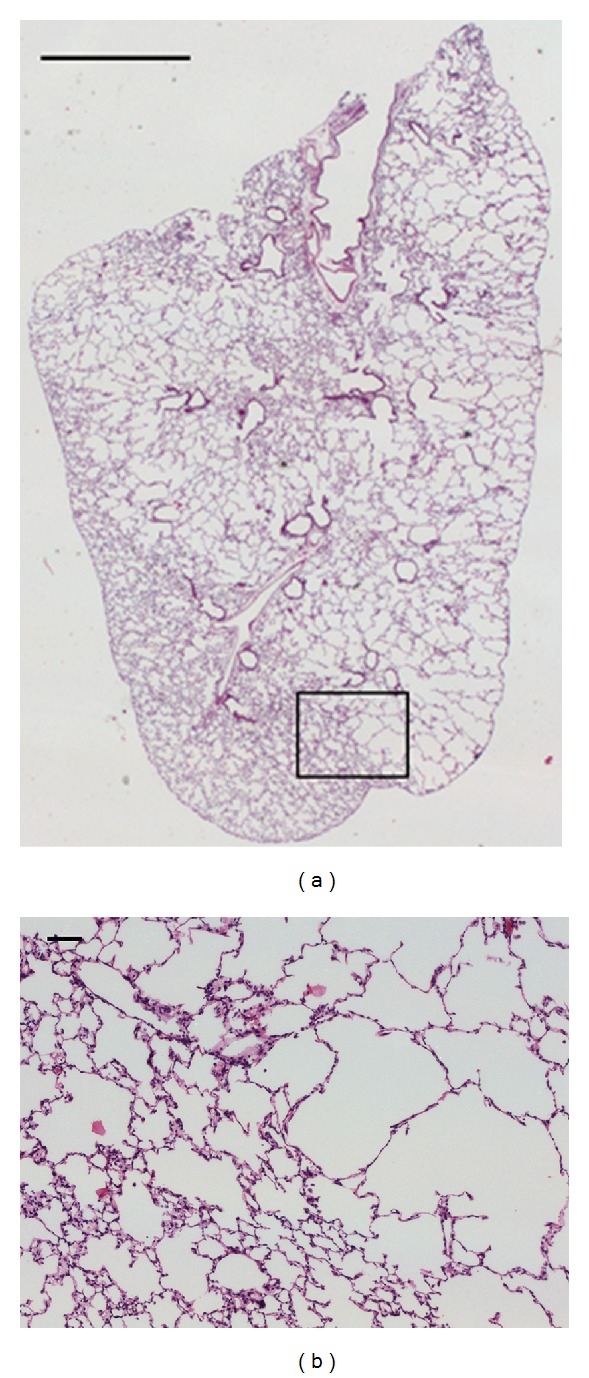
Sample histology images corresponding to a lung lobe slice of a mouse 17 days after instillation. (a) Mosaic acquired at 1.25x magnification. Scale bar represents 1000 microns length. (b) Zoomed area acquired at 10x. Scale bar represents 100 microns length. The mosaic (a) shows heterogeneous distribution of airspace enlargement. In the zoomed area (b), the left portion corresponds to normal alveolar tissue and the right shows the typical airspace enlargement found in emphysema.

Whole-slide views of all 1080 sections were acquired using an automated Axioplan 2ie Zeiss microscope (Carl Zeiss, Jena, Germany). Each slide was initially acquired with a Plan-Neofluar objective (numerical aperture NA = 0.035, magnification 1.25x, pixel resolution 3.546 *μ*m/pixel). The automatic threshold method proposed by Otsu [19] was then applied to detect all tissue areas. The size of the objects was measured and only objects with a reasonable size to represent entire sections of lung lobes were considered for further processing. For each object, a bounding box was created and the coordinates of its four vertices were sent to the microscope. Then, an automatic routine scanned those areas with a Plan-Neofluar objective (NA = 0.3, 10x, 0.725 *μ*m/pixel). Some overlap was allowed between image fields to facilitate the creation of large mosaics. The *Stitcher* ImageJ plugin [20] was used for it. The resulting mosaics were stored in a server for quantitative analysis.  Figure 3 shows a sample mosaic (Figure 3(a)) with a zoomed-in area showing heterogeneously distributed airspace enlargement (Figure 3(b)).

#### 2.3.2. Image Analysis

To accurately quantify bona-fide emphysematous air spaces, all vessels, alveoli, and spurious structures must be recognized and removed from the images because they might be confused with enlarged alveolar spaces. In the following paragraphs, we describe the method used to segment vessels and alveoli and the extraction of image descriptors.

**Segmentation**

**Figure 4:**
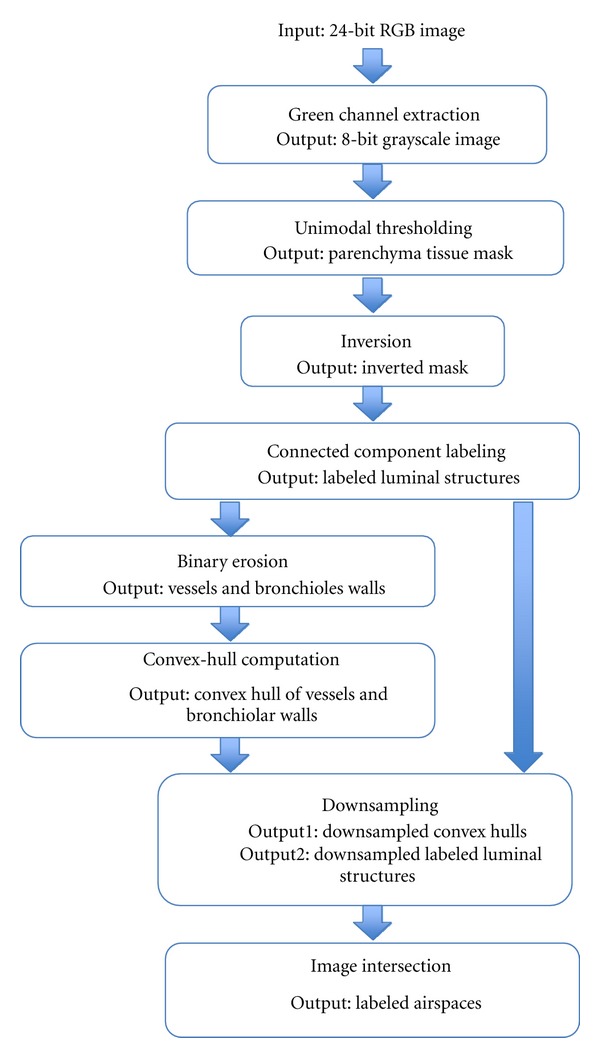
Flowchart of the airspace segmentation and labeling approach discarding the vessels and alveoli.

**Figure 5:**
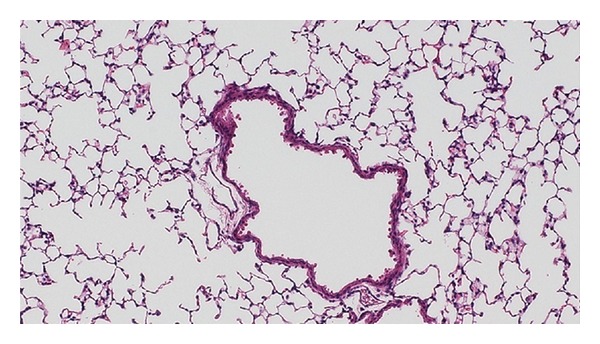
Lung parenchyma showing alveolar spaces and a large blood vessel. H&E staining.

**Figure 6:**
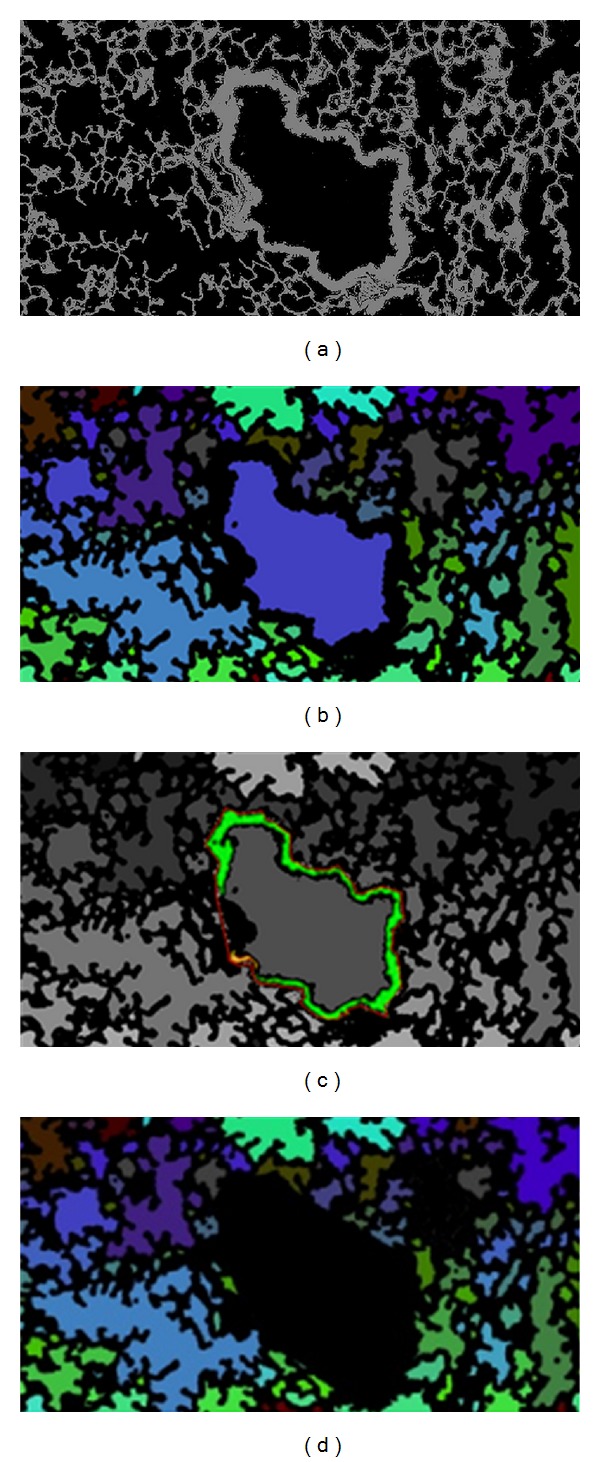
(a) Binarized image of H&E sample shown in Figure 4. (b) Image of the segmented alveoli calculated by connected component labeling. Notice how the large vessel in the center of the image is mistakenly included as airspace. (c) Vessel wall and its convex hull. (d) Removal of the vessel based on the intersection of the centroid of the vessel with the convex hull of the wall.

The main steps of the segmentation algorithm are summarized in the flowchart shown in Figure 4 and illustrated the snapshot shown in Figure 5. This image contains a vessel of a certain size in the center of the field of view. First, the 8-bit grayscale green channel was extracted from the 24-bit RGB, since it provides the greatest contrast between the background and the red-blue H&E stained tissue. Then, a mask of the parenchyma tissue was obtained by thresholding the histogram of the image. The histogram of a typical histological image is monomodal. Thus, common bimodal thresholding techniques—such as Otsu's thresholding—would not work well, leaving out some tissue structure walls. As an alternative, a maximum deviation unimodal thresholding [21] was used (Figure 6(a)) to extract all tissue areas from the background. The obtained mask was then inverted and all the luminal structures were segmented and labeled by connected component labeling (Figure 6(b)).

Since the lumen of blood vessels and bronchioles could be confused with enlarged airspaces, they must be detected and removed from the image before proceeding with the quantification. To this end, we applied binary erosion to the mask of the parenchyma using a structuring element of size 7 to remove all but the thickest walls, corresponding to vessels and bronchioles. Then, we used the geometric methods presented by [22, 23] and implemented in the Shapely Python package [24] to calculate the convex hull of the remaining walls (see Figure 6(c)). Next, the intersection between the convex hulls of the walls and the centroids of the labeled structures is performed and all the structures for which a nonempty intersection exists (i.e., vessels and bronchioles) are removed from the segmentation (see Figure 6(d)). At the end of this process, all the labeled regions represent airspaces.

The computation of the intersection is an intensive operation. To increase the speed, this operation was performed on a downsampled version of the image. The downsampling factor was empirically set to four to avoid loosing wall structures. However, the extraction of the airspace enlargement descriptors is performed on the full-resolution image.

**Extraction of Descriptors**

We extract four descriptors from the histology, namely, the linear mean intercept *L*_*m*_ and the moments of the airspace equivalent diameters (*D*_0_, *D*_1_, and *D*_2_).

The classical mean linear intercept *L*_*m*_ is defined as the mean length of the linear intercepts in the lung. In this paper, an approximation of *L*_*m*_ is computed as follows: the labeled image (Figure 6(d)) of each lobe is converted into an array of pixels; the image is then raster scanned, and the number of positive pixels between each pair of two consecutive null (wall) pixels is counted and its value (i.e., length of the *i*th linear intercept *l*_*i*_) is stored; then, the *L*_*m*_ is calculated as the mean of all the stored linear intercepts lengths on the lung lobe.

To compute the *D*_*v*_ indexes, we first calculate the area of the *i*th airspaces (*A*_*i*_) of the segmented, labeled image, by counting the number of pixels inside each labeled region. This value is scaled to physical dimensions using the objective calibration.

Then the equivalent airspace diameter of each space is defined as

(1) di=2Aiπ,

which is equal to the diameter of a circle with area *A*_*i*_.

The family of indexes *D*_*v*_ is then defined as the ratio of two moments of the equivalent airspace diameters distribution *d*:

(2) Dv=〈dv+1〉〈dv〉 with  v=0,1,2…,

where 〈⋯〉 indicates the arithmetic mean. *D*_*v*_ can be expressed as functions of the central moments of airspace diameters. In particular, *D*_0_ is the arithmetic mean of the airspace diameters. *D*_1_ is a function of its mean (*μ*) and variance (*σ* ^2^):

(3) D1=〈d2〉〈d1〉=μ(1+σ2μ2),

and *D*_2_ is a function of *μ*, *σ* ^2^, and skewness (*γ*) of the airspace diameters:

(4) D2=〈d3〉〈d2〉=μ[1+σ2μ2+σ2(2+σγμ)].

**Computational Architecture**

The code was written on a hybrid Python/C++ platform and was specifically designed to exploit parallelism. First, a process is spawn to walk the image directory tree and collect the files to be processed. Then, a job description is created for each image, which contains all the parameters used to guide the analysis through the steps detailed before. Each job is added to a shared queue on the network. Several processes access the remote queue from five different machines, download a job description, and execute it locally. Results are saved in a distributed file system.

#### 2.3.3. Statistics

The median and interquartile ranges (IQR) were calculated for each histology descriptor and time point. The control and elastase-treated groups were compared using the Mann-Whitney *U*-test. To measure progression, the same test was performed on the elastase-treated animals at successive time points. *P* values ≤ 0.01 were considered statistically significant. The *R* language and environment for statistical computing [25] were used for statistical analysis.

### 2.4. Classification

In the previous subsections, we have presented three different sets of disease descriptors: (1) micro-CT density-based descriptors: MLI (HU), VBT (%), RMBR (*μ*m), and LMBR (*μ*m); (2) Pulmonary Functional Test single compartment model parameters: Resistance (*R*) (cm H_2_O/mL/s) and Compliance (*C*) (mL/cm H_2_O); (3) RNA cytokine expression measured as concentration: IL6, IP10, KC, and MCP1. 

A Support Vector Machine (SVM) Classifier was created for each descriptor set. The chosen kernel was a Gaussian radial basis function. The classifier was defined by two parameters: the area of influence of the support vector on the data space *Υ* and the soft margin parameter *P*. The best parameter combination was selected by a grid search with exponentially growing sequences of *Υ* and *P*. In particular, *Υ* was chosen in the interval (10^−9^, 10^3^) and *P* in (10^−1^, 10^13^). Each combination of parameters was cross-validated, and the parameters with best cross-validation accuracy were selected. The dataset was divided in a training (60% of the samples) and test groups (the remaining 40%). 

For every classifier, a receiver operating characteristic (ROC) curve was generated. The histomorphometrical parameter *D*_2_ was used as the gold standard and the 3rd quartile value of the control animals was used as a threshold. For all possible combination of features, the classical performance indexes of Area Under the ROC Curve (AUC) and *f*_1_-score on the test dataset were computed. The results were then compared to determine the best classifier.

## 3. Results

### 3.1. Histological Analysis

**Figure 7:**
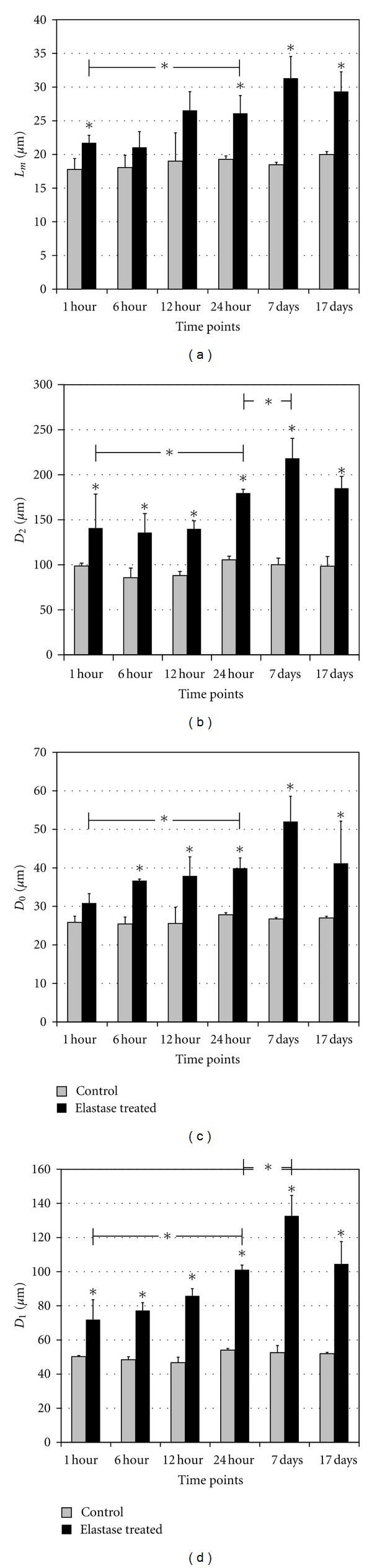
Evolution of *L*_*m*_ (a), *D*_2_ (b), *D*_0_ (c), and *D*_1_ (d) descriptors at different times after elastase instillation. *D*_2_ is the mean equivalent diameter, *D*_1_ and *D*_2_ are weighted indexes of airspace size distribution, and *L*_*m*_ is the mean linear intercept. Statistically significant differences between the control (*C*) and the elastase-treated (*E*) groups are indicated as well as differences between successive time points in the elastase group (*P* < 0.01,*).

The mean linear intercept (*L*_*m*_) and weighted mean (*D*_0_, *D*_1_, and *D*_2_) were computed and used as estimates of the airspace enlargement.  Figure 7 and Tables 2 (*L*_*m*_), 3 (*D*_0_), 4 (*D*_1_), and 5 (*D*_2_) show the progression of these parameters throughout the experiment for both groups (control and elastase induced). As expected, no progression was found in the control group, regardless of the parameter used. In the treatment group, all the parameters increased significantly (*P* < 0.01) from 1 hour to 24 hours after elastase treatment. *D*_2_ and *D*_1_ have also a statistically significant increase (*P* < 0.01) from 24 hours to one week after elastase treatment, which was somehow undetected by *L*_*m*_ and *D*_0_. No progression was detected in any case from one week to 17 days after the insult. 

Regarding the sensibility to differences between the control and treatment groups, *L*_*m*_ is significantly different at all time points except at 6 and 12 hours after elastase administration. *D*_0_  is significantly different at all time points except very early on, 1 hour after treatment. Contrarily, *D*_2_  and *D*_1_  are significantly different (*P* < 0.01) between treatment and control groups at all time points. In summary, although all the parameters yield similar results, *D*_2_  and *D*_1_  are more sensitive to differences between the emphysematous mice and controls.

The computation time per lung lobe section was about 20 minutes. 

### 3.2. Classification

We used three classifiers based, respectively, on: (1) Micro-CT density-based descriptors, (2) RNA cytokine expression measured as relative RNA concentration by qPCR, and (3) single compartment model parameters from pulmonary function tests.

**Figure 8:**
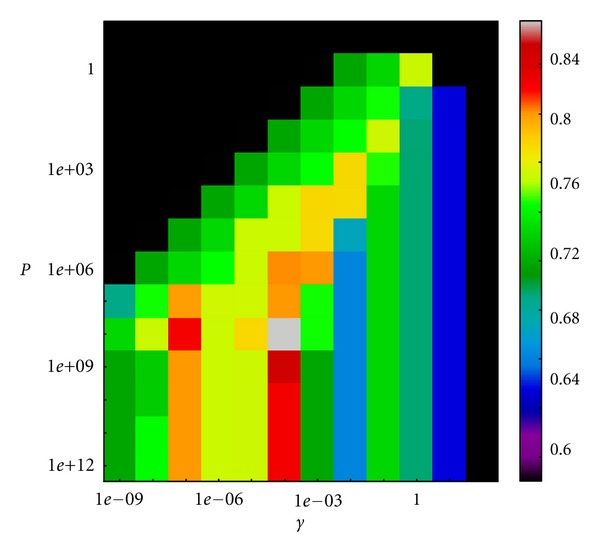
Cross-validation accuracy plot for the selection of the optimal work parameters of the micro-CT classifier (*Υ* = 10^−4^, *P* = 10^8^).

**Figure 9:**
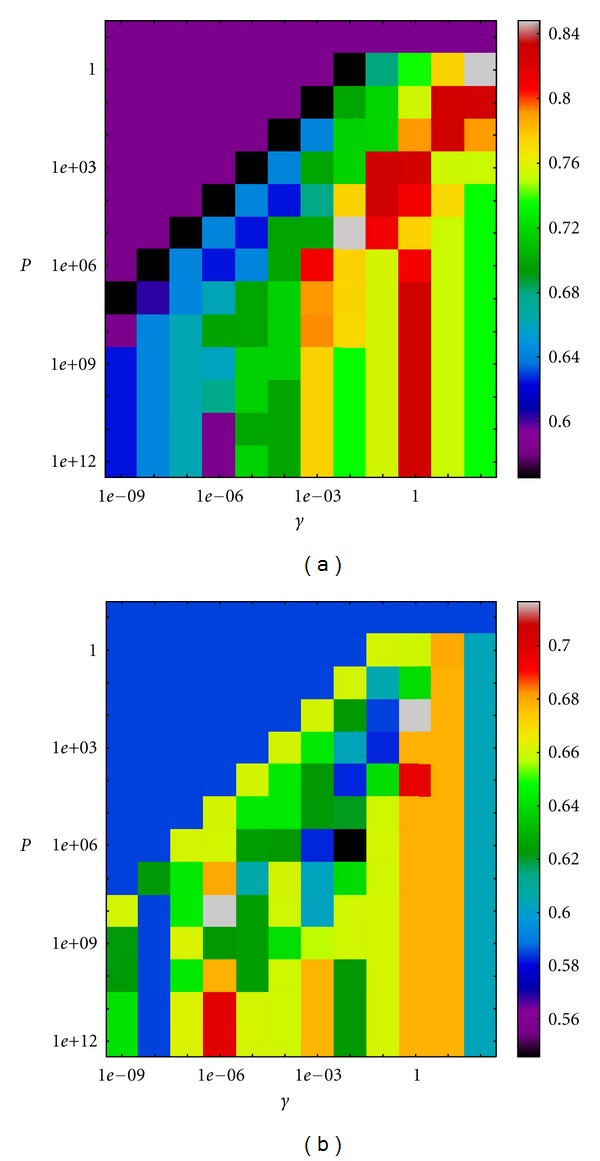
Cross-validation accuracy plot for the selection of the optimal work parameters for: (a) cytokine expression classifier (*Υ* = 10^−2^ and *P* = 10^5^); (c) pulmonary function tests classifier (*Υ* = 10^−6^ and *P* = 10^8^).

**Figure 10:**
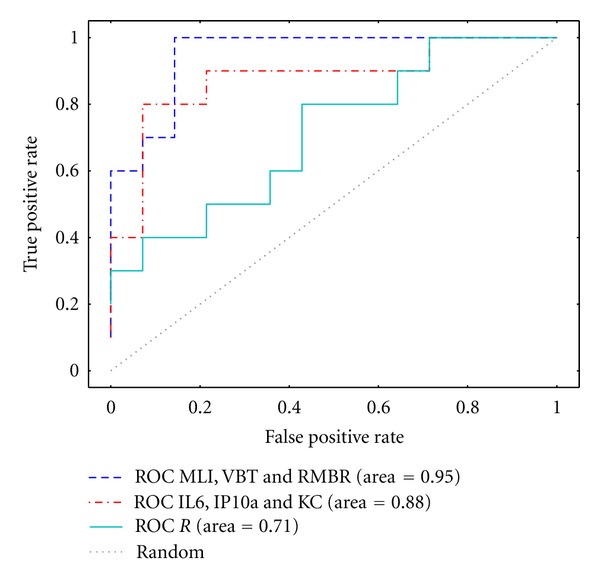
ROC curves of the classifiers tested on the best combination of features. The first set MLI, VBT, and RMBR were derived from micro-CT images. The second set corresponds to the RNA cytokine expression levels of IL6, IP10, and KC. The third set corresponds to the pulmonary functional parameter *R*. The features derived from micro-CT images show the best performance, because higher true positive rates can be obtained for the same false positive rate at most thresholds.

The cross-validation accuracy plots for the selection of the optimal estimated parameters are shown in Figures 8, 9(a) and 9(b) for the micro-CT, cytokine expression and pulmonary function tests classifier, respectively. The optimal estimated parameters, best set of features, AUCs, and *f*_1_-scores are given in Table 6 while Figure 10 shows the ROC curves for the best classifier of each parameter set. The Micro-CT-based classifier using as features MLI, VBT, and RMBR shows the best performance with an AUC of 0.95 and a *f*_1_-score of 0.92 and as shown by the ROC curve the highest true positive rates can be achieved for the same false positive rate. In general, RNA cytokine expression classifier using as features KC, IL6, and IP10 performs slightly worse than micro-CT with an AUC of 0.88 and *f*_1_-score of 0.71 although it achieves the best true positive rate of 0.8 for a false positive rate of 0.1. The classifier based on feature Functional resistance performs significantly worse than the other two classifiers with an AUC of 0.71 and an *f*_1_-score of 0.66, being the closest to the performance of a random classifier. 

## 4. Discussion

The main aim of this work was to evaluate and compare the sensitivity of the quantification of elastase-induced lung damage, using micro-CT-derived descriptors, pulmonary function tests based on a single compartment model, and RNA cytokine expression. Histomorphometry was used as gold standard for the comparison.

Our results show that *D*_2_ is able to distinguish between the control and the elastase-treated group at all time points, starting as early as one hour after treatment. This early airspace enlargement might be caused by surfactant dysfunction resulting from elastase administration. The ability of *D*_2_ to discriminate such early damage can be attributed to the fact that *D*_2_ heavily weights on enlarged airspaces and therefore, reflects better the airspace size distribution. In terms of the disease progression, *D*_1_ and *D*_2_ detected airspace enlargement during the first 24 hours and from that point until one week after treatment. This last increase was missed by *L*_*m*_ and *D*_0_.

Compared to previous studies, our histomorphometry values were obtained using a considerably larger sample size. In previous works, a few random fields were acquired from each mice lung. Here instead, mosaic images of whole lung lobe sections were acquired and analyzed, which was possible thanks to our fully automated software. Other interesting characteristics of automated systems like ours are the reduction of operator bias and a substantial reduction of the time dedicated to the analysis. Finally, the key for the success of our method is the fact that our toolset implements a mechanism to eliminate alveoli and vessels. Previously, major airways and vasculature were manually discarded at acquisition or analysis time using a great deal of manual interaction.

Micro-CT is especially appropriate to study *in vivo* the progression of animal models of pulmonary disease [13]. We have set up a generic protocol for micro-CT image acquisition that allows longitudinal studies [26]. The protocol includes endotracheal intubation and iso-pressure breath holds to reduce movement artifacts. Several segmentation and analysis methods were developed to quantify the effects of disease on the very noisy, artifact-plagued micro-CT images [12, 16]. These methods allow for quantitative measurements of the lungs and the airways separately, thus allowing to monitor disease development. In this work, we found that a SVM classifier using the micro-CT-derived, features MLI, VBT, and RMBR reached a high AUC and *f*_1_-score, thus indicating that micro-CT produces reliable measurements of airspace enlargement even at very early disease stages. Pulmonary function tests were also performed using forced oscillation techniques with endotracheal intubation. The SVM classifier trained on the Pulmonary function tests parameters using only tissue resistance (*R*) achieved the best AUC and *f*_1_-score. It could seem strange that the optimal classifier uses *R* instead of *C* or both. In long-term studies of the elastase-induced model, *C* gets clearly increased as a reflection of high lung stiffness. Our results presented in [12] show instead that *C* decreases during the first 24 hours. It is only at week 4 that *C* starts to increase. We hypothesize that this behavior may be related to the acute inflammatory reaction occurring in the elastase-induced model, starting immediately after treatment and nearly disappearing by day 7. On the other hand, the relatively good performance of the SVM classifier training on the cytokine expression levels could reflect this short-time inflammation. However, its ability to detect airspace enlargement in long-term studies has yet to be confirmed.

Finally, as explained in detail in the Results, the micro-CT-derived descriptors seem to be especially well suited for the estimation of airspace enlargement on the elastase-induced emphysema mice model.

## 5. Conclusion

In this paper, we presented open source software for the automatic quantification of airspace enlargement in large histological tissue sections. Using this software, we can automatically process large amounts of data in a relatively short period of time and with minimal user interaction. The automated measurements were able to detect airspace enlargement very early after the insult with elastase. Those measurements were used as ground truth to assess the sensitivity of micro-CT and other non-radiological techniques. Interestingly, typical respiratory-gated micro-CT density-based descriptors (mean lung density and relative volume below −900 HU) and the right radius of the mainstem bronchi achieved a high sensitivity and specificity discriminating early disease signs. 

## Figures and Tables

**Table 1:** Micro-CT data acquisition parameters.

X-ray source voltage	Current intensity	Exposure time per projection	Field of view
80 kVp	500 *μ*A	450 ms	43 × 43 mm

**Table 2:** Evolution of *L*_*m*_ at the experiment time points after elastase aspiration.

Time	Control		Elastase	
	Median (*μ*m)	IQR (*μ*m)	Median (*μ*m)	IQR (*μ*m)
1 hour	17.776	1.621	21.719	1.124
6 hours	18.061	1.802	21.054	2.336
12 hours	19.011	4.198	26.532	2.777
24 hours	19.269	0.517	26.110	2.641
7 days	18.478	0.341	31.327	3.216
17 days	19.987	0.427	29.348	2.926

IQR: interquartile range.

**Table 3:** Evolution of *D*_0_ at the experiment time points after elastase aspiration.

Time	Control		Elastase	
	Median (*μ*m)	IQR (*μ*m)	Median (*μ*m)	IQR (*μ*m)
1 hours	25.849	0.733	30.890	2.742
6 hours	25.458	1.865	36.686	0.428
12 hours	25.586	0.777	37.885	4.977
24 hours	27.838	0.973	39.903	2.689
7 days	26.751	2.795	52.024	6.578
17 days	27.008	1.046	41.181	10.946

**Table 4:** Evolution of *D*_1_ at the experiment time points after elastase aspiration.

Time	Control		Elastase	
	Median (*μ*m)	IQR (*μ*m)	Median (*μ*m)	IQR (*μ*m)
1 hour	50.220	0.688	71.907	11.718
6 hours	48.385	1.779	77.253	4.601
12 hours	46.708	3.180	85.817	4.261
24 hours	54.059	0.986	101.154	2.730
7 days	52.573	4.136	132.680	12.011
17 days	51.971	0.760	104.512	13.149

**Table 5:** Evolution of *D*_2_ at the experiment time points after elastase aspiration.

Time	Control		Elastase	
	Median (*μ*m)	IQR (*μ*m)	Median (*μ*m)	IQR (*μ*m)
1 hour	98.679	3.047	140.842	37.567
6 hours	85.693	10.657	135.809	21.120
12 hours	87.999	4.575	139.872	9.019
24 hours	105.524	4.026	179.636	4.278
7 days	100.105	7.213	218.298	22.146
17 days	98.459	10.738	185.006	13.165

**Table 6:** Optimal estimated parameters, best features and corresponding area under the ROC curve (AUC), and *f*_1_-score for each classifier (trained on micro-CT density-based descriptors, RNA cytokine expression data, and single compartment model parameters from pulmonary function tests (PFTs)) are shown.

Classifier	Optimal parameter	Features	AUC	*f*_1_-score
Micro-CT	Υ = 10^−4^; *P* = 10^8^	MLI, VBT, RMBR	0.95	0.92
Cytokine	Υ = 10^−2^; *P* = 10^5^	KC, IL6, IP10	0.88	0.71
PFTs	Υ = 10^−6^; *P* = 10^8^	*R *	0.71	0.66

## Abbreviations

AUC: Area under the ROC curve
B2M: Beta-2 microglobulin
*C*: Compliance
COPD: Chronic obstructive pulmonary disease
*D*_0_: Mean alveoli area
*D*_1_: *D*_0_ corrected by the first order statistical moment
*D*_2_: *D*_0_ corrected by the second order statistical moment
H&E: Hematoxylin and eosin
IL6: Interleukin 6
IP10: Immune protein 10
KC: Keratinocyte chemoattractant
LMBR: Left mainstem bronchus
*L*_*m*_: Linear intercept
Micro-CT: Microcomputed X-ray tomography
MLVI: Mean lung volume intensity
MCP1: Monocyte chemoattractant protein 1
PFT: Pulmonary function test
*R*: Lung resistance
RMBR: Right mainstem bronchus
ROC: Receiver operating characteristic
SVM: Support vector machine
VBT: Volume below threshold.
